# Degree of twist in the Achilles tendon interacts with its length and thickness in affecting local strain magnitude: a finite element analysis

**DOI:** 10.3389/fbioe.2024.1445364

**Published:** 2024-10-31

**Authors:** Shota Enomoto, Shunya Furuuchi, Tatsuki Ishibashi, Shu Yamada, Toshiaki Oda

**Affiliations:** ^1^ Institute for Promotion of Education and Campus Life, Okayama University, Okayama, Japan; ^2^ Graduate School of Science and Technology, Keio University, Yokohama, Japan; ^3^ Faculty of Science and Technology, Keio University, Yokohama, Japan; ^4^ Graduate School of Education, Hyogo University of Teacher Education, Kato, Japan

**Keywords:** achilles tendon, computational model, small composite design, subtendon, tendinopathy

## Abstract

**Introduction:**

The relationship between the twisting of the three subtendons of the Achilles tendon (AT) and local strain has received attention in recent years. The present study aimed to elucidate how the degree of twist in the AT affects strain using finite element (FE) analysis, while also considering other geometries (e.g., length, thickness, and width) and their combinations.

**Methods:**

A total of 59 FE models with different degrees of twist and geometries were created. A lengthening force (*z*-axis) of 1,000 N was applied to each subtendon (total: 3,000 N). The average value of the first principal Lagrange strain was calculated for the middle third of the total length of the model.

**Results:**

Statistical (stepwise) analysis revealed the effects of the degree of twist, other geometries, and their combinations on AT strain. The main findings were as follows: (1) a greater degree of twist resulted in higher average strains (*t* = 9.28, *p* < 0.0001) and (2) the effect of the degree of twist on the strain depended on dimensions of thickness of the most distal part of the AT (*t* = −4.49, *p* < 0.0001) and the length of the AT (*t* = −3.82, *p* = 0.0005). Specifically, when the thickness of the most distal part and length were large, the degree of twist had a small effect on the first principal Lagrange strain; however, when the thickness of the most distal part and length were small, a greater degree of twist results in higher first principal Lagrange strain.

**Conclusion:**

These results indicate that the relationship between the degree of twist and local strain is complex and may not be accurately assessed by FE simulation using a single geometry.

## 1 Introduction

The Achilles tendon (AT) is the strongest tendon in the human body. It has spring-like properties, stores and releases mechanical energy, and transmits the force created by the triceps surae muscles to the calcaneus, thereby enhancing the efficiency of body movements (Alexander and Bennet-Clark, 1977; Kawakami et al., 2002). However, the AT is constantly exposed to mechanical loads during physical activities and sports, increasing the likelihood of injuries, such as AT ruptures or tendinopathy.

Previous studies have indicated that the incidence of AT ruptures ranges from approximately 4.7 (Maffulli et al., 1999) to 37.3 per 100,000 individuals (Houshian et al., 1998). Furthermore, more than half of elite runners experience Achilles tendinopathy during their lifetime (Kujala et al., 2005). In addition, AT ruptures require 59–108 days (Metz et al., 2008) or an average of 6 months (Zellers et al., 2016) for the affected individual to return to work or play sports, depending on the treatment method. Achilles tendinopathy imposes long-term restrictions on sports participation (Habets et al., 2018). Therefore, elucidating the mechanisms underlying AT injuries and linking this knowledge to prevention is of great importance.

Although numerous studies have been conducted to elucidate the mechanisms underlying AT injury, the unique morphology of the AT complicates this issue. In recent years, attention has been focused on the relationship between injury and the unique composition of the AT, which, unlike many tendons, is composed of three independent subtendons arising from the lateral (LG) and medial (MG) heads of the gastrocnemius muscle and the soleus muscle (SOL) (Edama et al., 2015). In addition, cadaveric studies have shown that these subtendons are twisted with each other (Edama et al., 2015; Pękala et al., 2017). This twisted structure occurs without exception; moreover, the direction of the twist is consistent: when viewed from the proximal end, the right AT twists in a counterclockwise direction, whereas the left AT twists in a clockwise direction (Edama et al., 2015). Interestingly, the degree of twist in the AT varies among individuals, and previous studies have utilized this variation to categorize tendons into three groups (Pękala et al., 2017). Previous studies have suggested that local deformation, especially the distribution of non-uniform strains, contributes to the occurrence of tendon injuries (Enomoto and Oda, 2023; Maganaris et al., 2004). Consequently, recent studies have employed finite element (FE) analysis to investigate the relationship between the degree of twist and local strain (Diniz et al., 2024; Funaro et al., 2022; Knaus and Blemker, 2021).

Knaus and Blemker (2021) created three simple models of the AT that differed only in the degree of twist and investigated the magnitude of strain when an equivalent load was applied to each model using FE analysis. Their results indicated that, in models with higher degrees of twist, the strain on the subtendon originating from the LG was reduced. Similarly, Funaro et al. (2022) constructed three FE models that differed only in the degree of twist based on the geometry obtained from three-dimensional (3D) ultrasound and simulated the magnitude of local strain during exercises used in rehabilitation. The results revealed that models with less twisting exhibited higher peak strains. Although FE analysis has been used to investigate the relationship between the degree of twist and strain, these studies used only one original geometry and differences in geometry besides twisting were not considered. Because the magnitude of strain within the AT varies with geometry (Enomoto and Oda, 2023), FE analysis that investigated the strain when only the degree of twist varied left the following questions unanswered: 1) How does the combination of the degree of twist and other variations in geometry (e.g., length, thickness, and width) affect the strain? 2) Are there any interactions between these factors? Therefore, investigating the effects of the combinations of geometries and their interactions may provide deeper insights into the relationship between the degree of twist and strain.

The present study aimed to elucidate how the degree of twist in AT affects strain using FE analysis, while also considering other geometric properties and their combinations. However, considering that the combination of geometries significantly increases the number of geometric models, the study design combined FE analysis with the statistical method of a small composite design. Moreover, this study used many models with various combinations of geometries. Therefore, a simple, artificially created 3D model that allows for the easy modification of geometries was used. It was hypothesized that there were interactions between the degree of twist and other geometries in terms of their impact on the strain.

## 2 Materials and methods

### 2.1 Model geometries

The 3D AT geometry was created based on our previous study (Enomoto and Oda, 2023) using computer-aided design (CAD) software, Fusion 360 (Autodesk Inc., San Francisco, CA, United States). They reported that the AT model had the following eight parameters: thickness and width of the most proximal part, the minimum cross-sectional area (mCSA) part, and the most distal part, as well as the length and position of the mCSA. In this study, a CAD model that included eight parameters but not divided into subtendons was first created.

**FIGURE 1:**
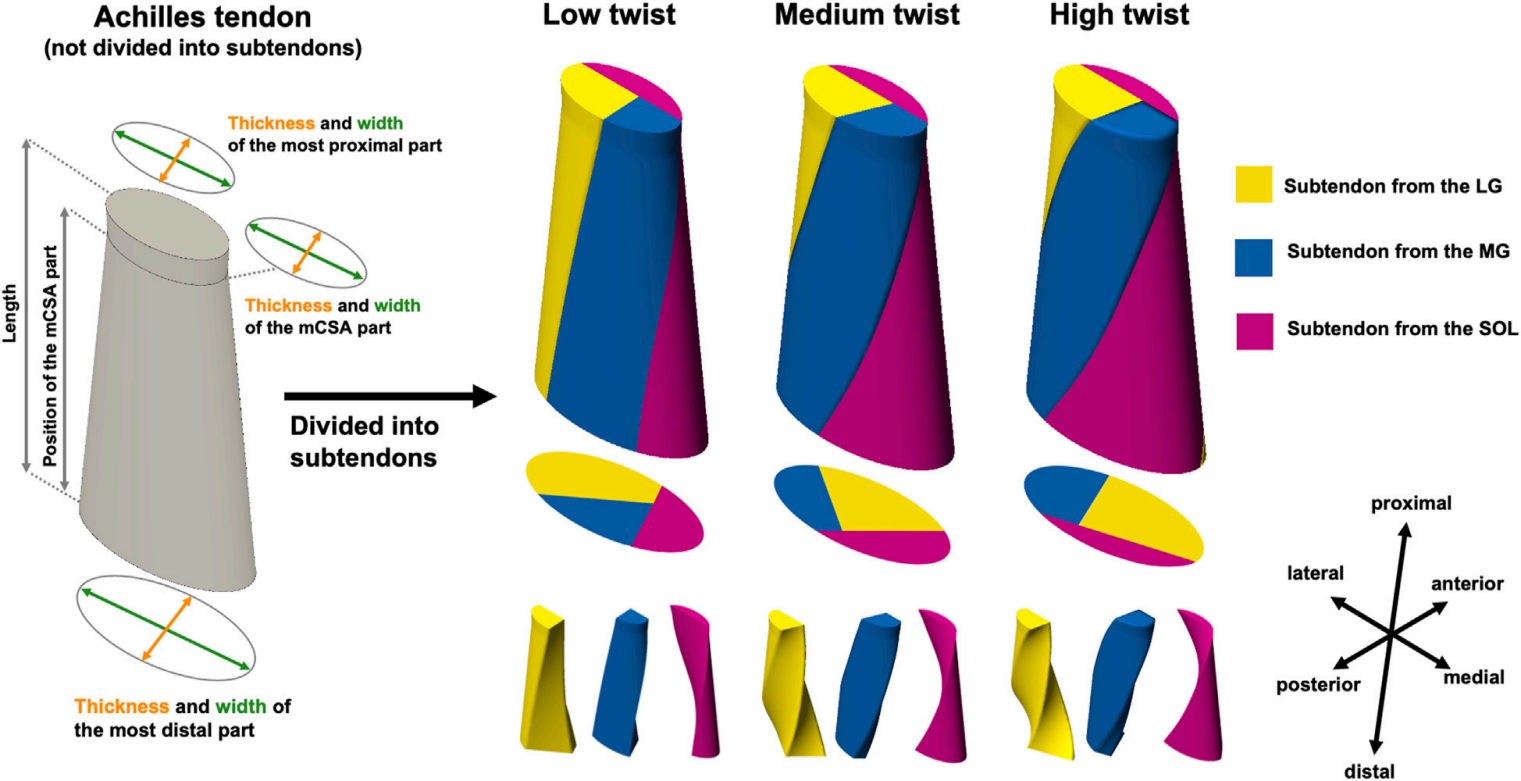
Overall view of the three-dimensional (3D) model of the Achilles tendon (AT) before being divided into subtendon and AT with three different degrees of twist of the subtendons. The 3D AT model before being divided into subtendon has eight parameters (thickness and width of the most proximal part, the minimum cross-sectional area [mCSA] part, and the most distal part, as well as the length and position of the mCSA), as reported in a previous study (Enomoto and Oda, 2023). 3D models of the AT with different degrees of twist of the lateral (LG) and medial (MG) heads of the gastrocnemius muscle and the soleus muscle (SOL) subtendons were created by subdividing the 3D AT model before dividing the subtendon using two internal surfaces.

Based on the classification of the average degree of AT twist reported by Pękala et al. (2017) (Type 1, 2, and 3), the CAD AT model was divided into three subtendons: LG, MG, and SOL. The division into subtendons was performed using two internal surfaces, and models with varying degrees of twist were created by utilizing the internal surfaces at different angles. In creating the internal surfaces, straight lines were first placed on the proximal and distal surfaces of the CAD AT model to define the cross-sections of the subtendons. Afterward, the internal surfaces were created by smoothly connecting the corresponding straight lines on the proximal and distal surfaces. The positions of the straight lines defining the subtendon cross-sections on the proximal and distal surfaces were determined with reference to the schematic diagrams at the point of the musculotendinous junction and the superior border of the insertion into the calcaneal bone for each twist type reported by Pękala et al. (2017). The degrees of twist in the models created based on Types 1, 2, and 3 reported by Pękala et al. (2017) were classified as low, medium, or high, respectively. The actual values of the degrees of twist are presented in the next paragraph. As mentioned above, our 3D AT model was idealized with nine parameters, including the degree of twist and eight other geometric parameters (Figure 1). The fibers of each subtendon were defined using FEBio Studio (Maas et al., 2012) and oriented from the proximal to distal surfaces. The procedure for creating these model shapes was similar to that used in previous studies (Funaro et al., 2022; Knaus and Blemker, 2021). Furthermore, for subsequent analysis, the angle formed by the corresponding proximal and distal edges of the internal surface was referred to the “*twist angle*”. Regarding the *twist angle,* the average value of the two internal surfaces was taken as the representative value.

To confirm whether the geometry reported in previous studies was accurately reproduced, the ratio of the cross-sectional area (CSA) of the subtendons to the total CSA, as well as the degree of twist in the models with only variations in the twist (low, medium, and high), was compared with the results of previous studies. The percentages of LG, MG, and SOL subtendons in the CSA of the external AT have been reported to be 43.59% ± 12.35%, 28.04% ± 10.04%, and 28.37% ± 9.78%, respectively (Pękala et al., 2017). Their values in the low-, medium-, and high-twist models, in which only the twist varied, were within the mean ±1 standard deviation (SD) reported in the previous study: the low-twist model, 44.05% for the subtendon from the LG, 27.89% for the subtendon from the MG, and 28.06% for the subtendon from the SOL; the medium-twist model, 43.13% for the subtendon from the LG, 29.05% for the subtendon from the MG, and 27.82% for the subtendon from the SOL; and the high-twist model, 47.51% for the subtendon from the LG, 31.19% for the subtendon from the MG, and 21.30% for the subtendon from the SOL. The twist angles were 59.50° in the low-twist model, 117.85° in the medium-twist model, and 152.25° in the high-twist model. These results are consistent with the relationships between twist angles and AT twist classifications reported in previous studies (Pękala et al., 2017).

FE analysis considering combinations of three levels of twist (low, medium, and high) and three levels (mean and mean ±1 SD) of the other eight geometric properties was conducted. The total number of factor combinations was 19,683 (3^9^), making it impractical to create and compute all models. Therefore, a simulation design was established using a small composite design, as outlined in the “*Design of simulation experiments*” section. The nine parameters (thickness and width of the most proximal part, thickness and width of the mCSA part, thickness and width of the most distal part, length and position of the mCSA part, and degree of twist) were designated as $x_{1},x_{2},\ldots ,x_{9}$ . The correspondences between the parameters and variables are presented in Table 1.

**TABLE 1:** Correspondence between variables and parameters.

Variable	Parameter
$x_{1}$	Thickness of the most proximal part
$x_{2}$	Width of the most proximal part
$x_{3}$	Thickness of the mCSA part
$x_{4}$	Width of the mCSA part
$x_{5}$	Thickness of the most distal part
$x_{6}$	Width of the most distal part
$x_{7}$	Length
$x_{8}$	Position of the mCSA part
$x_{9}$	Degree of twist

mCSA: minimum cross-sectional area.

### 2.2 Material properties and boundary conditions

In the present study, each subtendon was modeled as an incompressible transversely isotropic hyperelastic material (Weiss et al., 1996). The study employed the constitutive models implemented in FEBio Studio as “*trans iso Mooney-Rivlin*” (Maas et al., 2012), as described below (Funaro et al., 2022). The uncoupled strain-energy function is expressed as in Equation 1.

$$\Psi =F_{1}\left({\tilde{I}}_{1},{\tilde{I}}_{2}\right)+F_{2}\left(\tilde{\lambda }\right)+\frac{K}{2}{\left[\ln\left(J\right)\right]}^{2}$$ (1)

where, ${\tilde{I}}_{1}$ *and* ${\tilde{I}}_{2}$ represent the first and second invariants, respectively, of the deviatoric variant of the right Cauchy-Green deformation tensor; $\tilde{\lambda }$ represents the deviatoric part of the stretch along the fiber direction; and *J =* det (F) represents the Jacobian of the deformation. $F_{1}$ is the material response of the isotropic ground substance matrix, and $F_{2}$ is the contribution from the fiber family. $F_{1}$ is described as a neo-Hookean model and corresponds to $\frac{C_{1}\left(I_{1}-3\right)}{2}$ . The resulting fiber stress is expressed as in Equation 2.

$$\tilde{\lambda }\frac{\partial F_{2}}{\partial \tilde{\lambda }}=\left\{\begin{array}{l} 0\\ C_{3}\left(e^{C_{4}\left(\tilde{\lambda }-1\right)}-1\right)\\ C_{5}\tilde{\lambda }+C_{6} \end{array}\right.\begin{array}{c} \;\tilde{\lambda }\leq 1\\ 1<\tilde{\lambda }<\lambda_{m}\\ \;\tilde{\lambda }\geq \lambda_{m} \end{array}$$ (2)

where, $\lambda_{m}$ represents the stretch at which the fibers are straightened; $C_{3}$ and $C_{4}$ are the scaling of the exponential stresses and rate of uncrimping of the fibers, respectively; $C_{5}$ represents the modulus of the straightened fibers; and $C_{6}$ is derived from the requirement that the stress is continuous at $\lambda_{m}$ . The values for each material property were obtained from Hansen et al. (2017) and Knaus and Blemker (2021), which were similar to those reported by Funaro et al. (2022), as shown below: $C_{1}$ = 46.52 MPa, $C_{2}$ = 0, $C_{3}$ = 19.4 MPa, $C_{4}$ = 26.80, $C_{5}$ = 928 MPa. $\lambda_{m}$ was set to 1.03.

The proximal surface of each subtendon was displaced only in the longitudinal direction (*z*-axis). A lengthening force (*z*-axis) of 1,000 N was applied to each subtendon (total: 3,000 N). The total lengthening force of 3,000 N is an approximation of the maximum AT force during plantar flexor maximal voluntary isometric contraction (2,714.8N; Geremia et al. (2018)); following Yin et al. (2021), this lengthening force was evenly distributed across each subtendon. The distal surface was fixed. Additionally, the interfaces between the subtendons were modeled as frictionless contacts and separation was not considered (Diniz et al., 2023). All simulations were performed quasi-statically using FEBio Studio (Maas et al., 2012).

### 2.3 Mesh convergence test

The model was meshed with tetrahedral second-order elements. Mesh convergence analysis was performed by running repeated simulations on models with different mesh sizes, low twisting, and other unchanged geometries. The mesh was considered sufficiently refined when reductions in mesh size resulted in less than a 5% change in the first principal Lagrange strain (Enomoto and Oda, 2023; Jones and Wilcox, 2008). Consequently, the numbers of elements, nodes, and average maximum element edge length in this model were 24,802, 49,386, and 1.74 mm, respectively (Supplementary Figure S1).

### 2.4 Design of simulation experiments

Assuming a second-order model as shown below, where the response variable y is the strain and the factors are $x_{1},x_{2},\ldots ,x_{9}$ , the magnitudes of the main effects and interactions were quantitatively evaluated.

$$y=\beta_{0}+\beta_{1}x_{1}+\beta_{2}x_{2}+\cdots +\beta_{9}x_{9}+\beta_{12}x_{1}x_{2}+\beta_{13}x_{1}x_{3}+\cdots$$

$$+\beta_{89}x_{8}x_{9}+\beta_{11}x_{1}^{2}+\beta_{22}x_{2}^{2}+\cdots +\beta_{99}x_{9}^{2}+\varepsilon$$

In this second-order model, $\beta_{i}$ and $\beta_{ii}$ represent the first- and second-order effects, respectively, of factor $x_{i}$ , where the sum of these effects is called main effects. Moreover, $\beta_{ij}$ denotes the two-factor interaction between $x_{i}$ and $x_{j}$ , while ε represents the error. By estimating $\beta_{i},{\;\beta }_{ii},$ and $\beta_{ij}$ from the simulation, the relationship between the response y and factors $x_{1},x_{2},\ldots ,x_{9}$ can be quantitatively determined. This allows for the visualization of changes in response y when $x_{i}$ increases by one unit or the interactions between $x_{i}$ and $x_{j}$ vary, thereby providing valuable information. Central composite designs are often used in experimental designs based on second-order models (e.g., Myers et al., 2016). In the present study, to estimate the effects of the nine factors using fewer simulation runs, a small composite design was employed (Draper and Lin, 1990). The levels for each factor were set at the mean and mean ± 1 SD. However, for the position of the mCSA part, the levels were set such that the ratios of the lengths from the fixed plane were evenly spaced after logit transformation. Following a small composite design, a two-level factorial design required 20 runs as per the Plackett-Burman design and axial points were set at the original measurement’s mean ± 1 SD. Moreover, because this was a simulation study and repetitions were not necessary, the number of repeats at the center point was set to one. Moreover, for the *twist angle*, models were created with three different degrees of twist (low, medium, and high) and the measured values of the actual *twist angle* from the models were used for analysis. Consequently, 59 FE models were created. The assignment of each parameter for all the models is shown in Supplementary Table S1.

### 2.5 Strain analysis

The distribution of non-uniform strains within tendons has been shown to contribute to the occurrence of tendon injuries (Enomoto and Oda, 2023; Maganaris et al., 2004). Therefore, the first principal Lagrange strain was calculated to quantify the strain within the AT during loading. Based on Funaro et al. (2022), the average value of the first principal Lagrange strain was calculated for the middle third of the total length of the model.

### 2.6 Validation

The validity of the model in this study was examined by comparing the amount of strain and the force-elongation relationship in the model with only variations in the twist (low-, medium-, and high-twist models), as per experimental results obtained in a previous study (Kongsgaard et al., 2011). Specifically, the strain and force-elongation relationship of the model under load in this study were compared with those reported in a previous study during isometric plantar flexion (Kongsgaard et al., 2011). Graphical data in the previous study were extracted using WebPlotDigitizer (Rohatgi, 2017).

### 2.7 Analysis of FE simulation

Based on the data collected using a small composite design, the parameters of the second-order model were estimated using the stepwise regression method with both entry ( $\left.P_{in}\right)$ and exit ( $P_{out}$ ) values set to 0.05 to identify main effects and interactions, while considering the effects of the heredity principle, including the parent main effects for significant interactions. Additionally, to eliminate the influence of the units of factors $x_{1}$ to $x_{9}$ , each factor was standardized to have a mean of 0 and a standard deviation of 1.

## 3 Results

### 3.1 Validation

**FIGURE 2:**
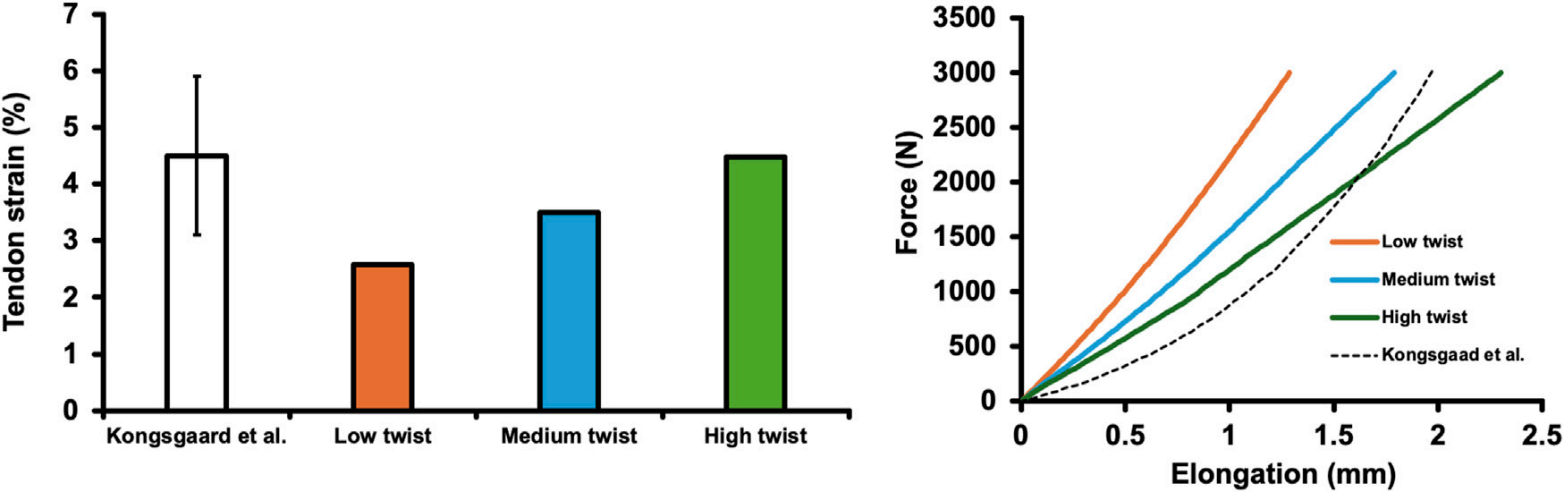
Achilles tendon strain of the finite element models varying only in the degree of twist (low-, medium-, and high-twist models) and that reported in a previous study (Kongsagaad et al., 2011) (at a load of approximately 2,000 N) (left). The force-elongation relationship of finite element models varying in the degree of twist (low-, medium-, and high-twist models) and that reported in a previous study (Kongsagaad et al., 2011) (right).

Previously, Kongsgaard et al. (2011) reported a maximum AT force of 2,011 N during plantar flexor maximal voluntary isometric contraction, with an AT strain of 4.5% ± 1.4%. In this study, the strains observed in the low-, medium-, and high-twist models, varying only in twist (at a load of approximately 2,000 N), were 2.58%, 3.50%, and 4.48%, respectively, which were approximately within the 1 SD reported in previous studies (Figure 2). Regarding the force-elongation relationship, although it did not show as distinct a nonlinearity, as that reported by Kongsgaard et al. (2011), the portion of the force-elongation relationship above approximately 2,000 N reported by Kongsgaard et al. (2011) fell within the range of this study (Figure 2).

### 3.2 Example results of the simulation

**FIGURE 3:**
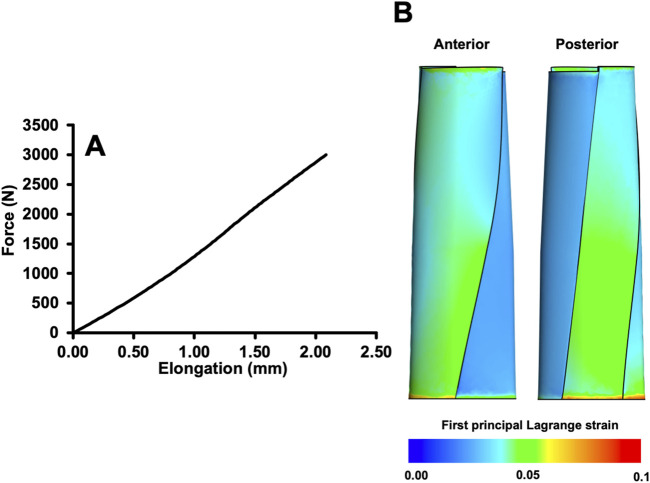
Force-elongation relationship **(A)** and distribution of the first principal Lagrange strain **(B)** when a total load of 3,000 N was applied to the example model (ID11).

The simulation results for ID 11 (the assignment of each parameter for the models is shown in the Supplementary Table S1) showed a maximum elongation of 2.08 mm (Figure 3A), and the average first principal Lagrange strain was 0.0383 (Figure 3B).

### 3.3 Models with varying degrees of twist only

**FIGURE 4:**
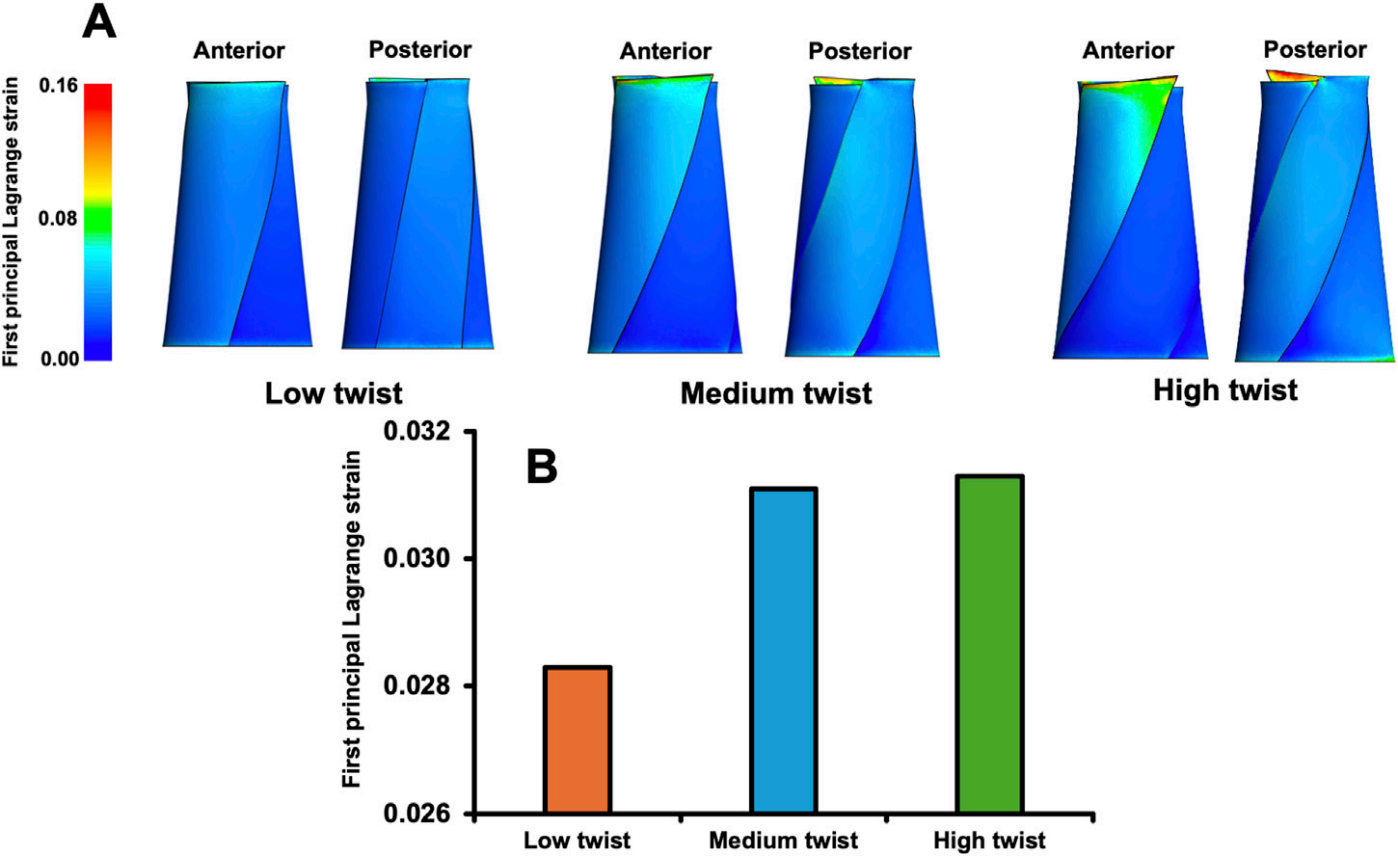
Distribution of the first principal Lagrange strain when a total load of 3,000 N was applied to finite element models that varied only in the degree of twist **(A)**; the average first principal Lagrange strain in the middle third of these models **(B)**.

The average first principal Lagrange strain in the medium-twist model (0.0311) and the high-twist model (0.0313) were 9.9% and 10.6%, respectively, greater than that in the low-twist model (0.0283) (Figure 4). However, the average first principal Lagrange strains of the medium- and high-twist models were similar (0.6%) (Figure 4).

### 3.4 Stepwise regression method

**FIGURE 5:**
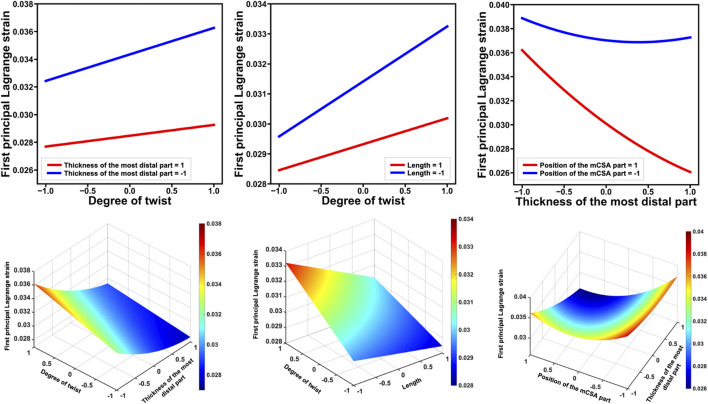
The top line displays the factors that showed significant interactions with the degree of twist regarding their effects on the first principal Lagrange strain, as well as those that exhibited significant interactions and had the highest absolute *t*-value. The bottom line shows these results in a surface plot. Each factor was standardized to have a mean of 0 and a standard deviation of 1. For example, the left figure in the top line shows the first principal Lagrange strain on the vertical axis and the degree of twist on the horizontal axis, with thickness of the most distal parts at −1 and 1. The fact that these lines are not parallel indicates that the additivity of the effect does not hold and that an interaction between two factors exists. When the thickness of the most distal part = 1, the degree of twist has a small effect on the first principal Lagrange strain, whereas when the thickness of the most distal part = −1, the degree of twist has a greater effect on the first principal Lagrange strain. mCSA: minimum cross-sectional area.

**FIGURE 6:**
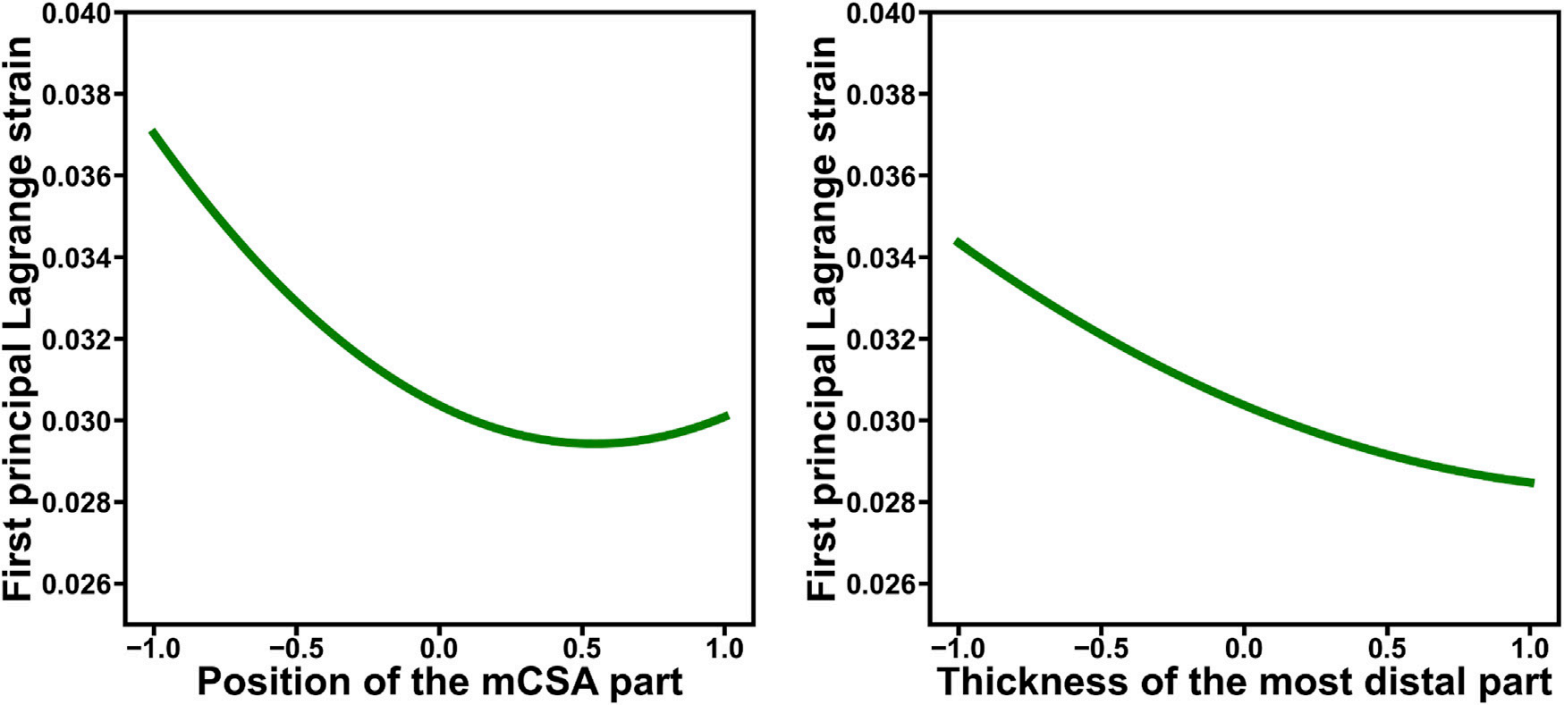
The composite functions of the main effects for parameters that exhibited significant main (first- and second-order) effects on the first principal Lagrange strain, as identified through stepwise analysis, are demonstrated. Each factor was standardized to have a mean of 0 and a standard deviation of 1. For example, the left figure demonstrates that the relationship between the strain and the position of the minimum cross-sectional area (mCSA) is not just a linear relationship but that there is a position of the mCSA value at which the strain is minimized.

Stepwise analysis revealed the complex effects of the degree of twist, other geometric properties, and their combinations on the AT strain (Table 2). Significant first-order effects were observed for factors except for the thickness ( $x_{1}$ ) and width ( $x_{2}$ ) of the most proximal part (*t* = −25.51 to 9.28, *p* < 0.0001). Additionally, interactions were found among many factors (*t* = −15.92 to 4.35, *p* < 0.05). Regarding the interaction, the greatest effect was between the thickness of the most distal part ( $x_{5}$ ) and position of the mCSA part ( $x_{8}$ ) (*t* = −15.92, *p* < 0.0001) (Figure 5). The thickness of the most distal part ( $x_{5}$ ) (*t* = −4.49, *p* < 0.0001) and length ( $x_{7}$ ) (*t* = −3.82, *p* = 0.0005) showed interactions with the degree of twist in their effects on the first principal Lagrange strain (Figure 5). Specifically, when the thickness of the most distal part and length were large, the degree of twist had a small effect on the first principal Lagrange strain; however, when the thickness of the most distal part and length were small, a greater degree of twist resulted in higher first principal Lagrange strain. In addition, significant second-order effects were found for the thickness of the most distal part ( $x_{5}$ ) (*t* = 2.80, *p* = 0.008) and position of the mCSA ( $x_{8}$ ) (*t* = 8.54, *p* < 0.0001) (Figure 6).

**TABLE 2:** Significant main effects, and interaction effects that were identified using a stepwise regression.

Variable	Estimated value	*t*-value	*p*-value
$x_{2}$	2.25	1.74	0.0907
$x_{3}$	−22.34	−16.05	<.0001
$x_{4}$	−19.79	−14.10	<.0001
$x_{5}$	−29.36	−21.60	<.0001
$x_{6}$	−15.49	−10.91	<.0001
$x_{7}$	−10.46	−7.61	<.0001
$x_{8}$	−34.69	−25.51	<.0001
$x_{9}$	13.49	9.28	<.0001
$x_{2}\times x_{8}$	3.47	2.84	0.0071
$x_{3}\times x_{4}$	3.61	2.78	0.0084
$x_{3}\times x_{5}$	2.75	2.28	0.0280
$x_{3}\times x_{7}$	2.54	2.03	0.0495
$x_{3}\times x_{8}$	5.30	4.35	0.0001
$x_{4}\times x_{8}$	4.05	3.32	0.0020
$x_{5}\times x_{8}$	−21.36	−15.92	<.0001
$x_{5}\times x_{9}$	−5.66	−4.49	<.0001
$x_{6}\times x_{8}$	−8.92	−6.65	<.0001
$x_{7}\times x_{9}$	−4.84	−3.82	0.0005
$x_{5}^{2}$	10.45	2.80	0.0080
$x_{8}^{2}$	31.90	8.54	<.0001

The estimated values presented in this table were multiplied by 10^4^ to enhance the readability. This table should be referred to together with Table 1, which shows the correspondence between variables and parameters.

## 4 Discussion

This study elucidated how the degree of twist in AT affects the strain, while also considering other geometric properties and their combinations, using FE analysis. The results revealed complex relationships between strain and geometry, including the main effects of each factor, and interactions between factors. Although greater degrees of twist resulted in increased strain, it was evident that the degree of twist interacted with other geometric properties in terms of its impact on the strain. Thus, the relationship between the degree of twist and strain is affected by other geometric properties.

The results of this study revealed that factors, except the thickness ( $x_{1}$ ) and width ( $x_{2}$ ) of the most proximal part, had significant effects on the strain (Table 2). Additionally, the first-order effects of all factors, except the width of the most proximal part ( $x_{2}$ ) and the degree of twist ( $x_{9}$ ), were negative, indicating that smaller values of these factors were associated with a higher strain. A previous study using FE methods also reported that smaller dimensions (thinner, narrower) of AT parts resulted in higher local strains (Enomoto and Oda, 2023), which is consistent with our findings. Furthermore, the first-order effects showed that the position of the mCSA part ( $x_{8}$ ), the thickness of the most distal part ( $x_{5}$ ), and the thickness ( $x_{3}$ ) and width ( $x_{4}$ ) of the mCSA part ranked in the top four in terms of *t*-values, suggesting that changes in these parameters have a large impact on the strain. Enomoto and Oda (2023), although the variation of the position of the mCSA part was not considered, reported that reducing the thickness of the most distal part and the thickness and width of the mCSA part greatly increases local strain, which aligns the findings of current study with the previous study.

In this study, the *t*-value for the first-order effect of the twist ( $x_{9}$ ) was positive (Table 2), indicating that a greater degree of twist resulted in higher average strains. This was also evident in the results of the models that varied only in the degree of twist (Figure 4). Funaro et al. (2022) investigated the magnitude of the strain experienced by the AT during various rehabilitation exercises using FE analysis, considering the degree of twist. Their findings suggested that the degree of twist did not affect the average strain in the middle third of the AT. These results differ from those of the present study. Figure 5, which illustrates the results of significant interactions in this study, shows that although the degree of twist had a small effect on the strain under conditions of greater thickness of the most distal part and length, an increased twist under conditions of lesser thickness of the most distal part and length resulted in a higher strain. These findings indicate that the relationship between the degree of twist and strain is affected by the dimensions of the other geometric properties. Funaro et al. (2022) reported a model length of 40 mm, which is larger than the length used in this study when the length was not varied (35.5 mm: mean ±0 SD, Supplementary Table S1). Therefore, considering the interaction between the degree of twist and length observed in this study, it is possible that the relatively larger model length in Funaro et al.‘s study may have prevented the degree of twist from affecting the magnitude of the average strain. Meanwhile, although the thickness of the most distal part in Funaro et al. (2022) was not reported, it is also possible that its relatively large size similarly prevented the degree of twist from affecting the magnitude of the average strain. Consequently, the geometric properties that interact with the degree of twist in its effect on strain may explain the discrepancies between a previous study and the present study.

Additionally, Knaus and Blemker (2021) used FE analysis to demonstrate that AT models with a higher degree of twist exhibited reduced average strain in the subtendons originating from the LG, which contradicts the findings of this study. Regarding the boundary conditions, Knaus and Blemker (2021) applied pressure to induce a prescribed displacement in each subtendon. The displacement of the proximal surfaces of the MG, LG, and SOL subtendon were 7.6 mm 7.6 mm, and 5.9 mm, respectively. Thus, while the displacement was consistent across the models, the applied pressure was likely not uniform. Meanwhile, in this study, a constant lengthening force was applied to each subtendon, which was identical across all models. In other words, while the lengthening force was consistent across models, the displacement varied. In fact, the maximum displacement of the low- (1.29 mm) and high-twist models (2.30 mm) differed by approximately 1.8 times (Figure 2). For example, if the boundary conditions were standardized across models based on displacement, as in Knaus and Blemker (2021), the low-twist model would require a lengthening force greater than that required by the high-twist model in this study. As a result, the relative strain values, as well as the order of strain magnitude between these models, could change. These changes could alter the relationship between the degree of twist and strain magnitude. Therefore, the differences between the results of this study and those of Knaus and Blemker (2021) may be explained by differences in the boundary conditions.

However, each study differs in other various aspects, such as combinations of geometries, whether interactions are considered, methods for calculating strain, and the AT geometry used. The relationship between the degree of twist and the strain in this study was complex, involving interactions with multiple geometries. This relationship may also be sensitive to other factors that were not considered in this study. Future research will need to investigate the relationship between the twist and strain by considering many parameters and conditions. Thus, the experimental design methods have proven to be highly effective.

This study revealed interactions among many geometries, in addition to the twist (Table 2; Figure 5). Furthermore, the thickness of the most distal part ( $x_{5}$ ) and the position of the mCSA part ( $x_{8}$ ) exhibited second-order effects on strain (Table 2; Figure 6). These second-order effects indicate that, for example, the strain increases under conditions of both extremely large and small thicknesses of the most distal part, suggesting that there is an optimal dimension at which the strain is minimized. Previous studies using FE analysis to investigate the strain and stress distributions in AT have often employed a single geometry (Funaro et al., 2022; Handsfield et al., 2017; Knaus and Blemker, 2021; Diniz et al., 2023; 2024). Although some studies used multiple geometries, they did not consider factors such as twisting (Enomoto and Oda, 2023; Hansen et al., 2017) or the presence of subtendons (Shim et al., 2018); thus, they did not estimate interactions or second-order effects. By combining FE methods with a small composite design, this study provided complex and detailed information about the relationships among geometry, degree of twist, and strain.

Several researchers have studied the relationship between the degree of twist and AT injuries. In addition, previous studies have indicated that local deformations of the tendon, particularly non-uniform distributions of strain, are associated with the occurrence of injuries (Enomoto and Oda, 2023; Maganaris et al., 2004). Therefore, the results of this study suggest that a larger degree of twist, as affected by other geometric properties, may increase the likelihood of injury. Conversely, previous studies using FE analysis indicated that ATs with smaller degrees of twist may have a higher risk of injury (Funaro et al., 2022). Similarly, Knaus and Blemker (2021) suggested that more twisted ATs could potentially have a lower risk of injury. In addition, Shim et al. (2018) and Handsfield et al. (2020) reported that a moderate degree of twist in AT could increase its strength. In studies other than the FE analysis, it has been advocated that a high degree of twist can increase the risk of AT injuries due to increased vascular compression (Pękala et al., 2017) and internal tendon pressure (Pringels et al., 2023). Thus, reports on the relationship between the degree of twist and injuries are inconsistent, and it remains unclear whether high or low twisting is associated with AT injuries. Recently, attempts have been made to measure the degree of twist, which is considered difficult to measure *in vivo*, using high-field (7T) magnetic resonance imaging (Cone et al., 2023), and to estimate the degree of twist using ultrasound-derived AT displacement data (Lecompte et al., 2024). Future studies should comprehensively explore the relationship between the degree of twist and injury using *in vivo* and *in vitro* experiments and *in silico* simulations.

This study had several limitations. The study aimed to elucidate the effect of the degree of twist on the AT strain, considering combinations with other geometric properties. To achieve this, a simple and easily modifiable artificial geometry based on the measurement data was used for the simulations, and relatively simple loads were applied to each subtendon. Although this simple geometry and loads were sufficient to achieve the purpose of the study, future research should examine more detailed models that closely resemble *in vivo* AT geometries and loads. Furthermore, this study modeled each subtendon as an incompressible transversely isotropic hyperelastic material and did not consider variations in the mechanical properties of the subtendons. Meanwhile, a recent study has emphasized the importance of transverse poroelasticity in the material behavior of tendons (Safa et al., 2020). Future studies will need to consider this aspect when modeling the material properties of the AT. In addition, FE simulations that consider variations in mechanical properties would provide more detailed information on the relationship between twist and strain. Regarding the validity of this study, the validation of our model was limited to comparisons with strains and force-elongation relationships reported in previous experimental studies, and the validity of the local strain has not been verified. This is an issue that should be addressed in future research. Lastly, while the focus was on the magnitude of local strain, its distribution was not examined, as this point was not crucial to answering our research question. However, this should be considered in future studies.

## 5 Conclusion

Recently, the impact of the degree of twist on AT strain was investigated using FE analysis. However, previous studies were limited in that they used only one original geometry and differences in geometry other than twisting were not considered. This study revealed that how combinations of the degree of twist and other geometric properties affect the strain. The study was performed using artificially created 3D AT FE models and a small composite design. The results revealed that greater degrees of twist increased the strain in the middle third of the AT. Furthermore, the degree of twist was found to interact with the thickness of the most distal part and length in terms of its impact on the strain. Specifically, when the thickness of the most distal part and length were large, the degree of twist had a small effect on the strain; however, when the thickness of the most distal part and length were small, a greater degree of twist resulted in higher first principal Lagrange strain. These results indicate that the relationship between the degree of twist and strain is complex and may not be accurately assessed by FE simulation using a single geometry. More detailed 3D models of the AT and variations in the material properties of the subtendons may affect these relationships. Furthermore, these factors may affect not only the magnitude of local strain but also its distribution; thus, these should be investigated further in future studies.

## Data Availability

The raw data supporting the conclusions of this article will be made available by the authors, without undue reservation.
