# Supplementary material for: Degree of twist in the Achilles tendon interacts with its length and thickness in affecting local strain magnitude: a finite element analysis
Source: Front Bioeng Biotechnol. 2024 Oct 31;12:1445364. doi: 10.3389/fbioe.2024.1445364 (PMC11561387; doi:10.3389/fbioe.2024.1445364)
Supplement: Supplementary file 1 [file DataSheet1.docx]

Supplementary Materials

# Supplementary Figures and Tables

##
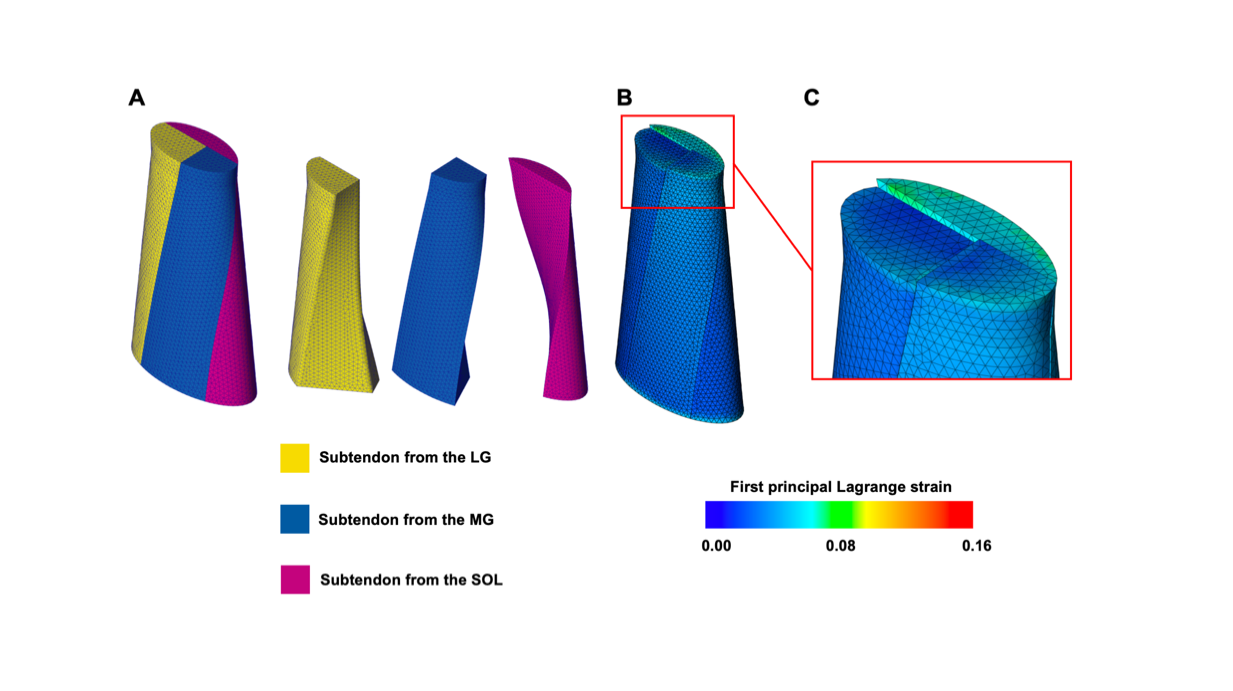
Supplementary Figure

**Supplementary Figure 1.** The mesh adopted as a result of the mesh convergence test (model with low twist and other unchanged geometries) (A), its mesh deformation and the distribution of the first principal Lagrange strain at the final state in the mesh convergence test calculation (B), and an enlarged view (C). LG: lateral head of the gastrocnemius, MG: medial head of the gastrocnemius, SOL: soleus.

## Supplementary Table

**Table** The assignment of each geometric parameter for all models

|  | Thickness of the most proximal part (mm) | Width of the most proximal part (mm) | Thickness of the mCSA part (mm) | Width of the mCSA part (mm) | Thickness of the most distal part (mm) | Width of the most distal part (mm) | Length (mm) | Position of the mCSA part (%) | Degree of twist |
| --- | --- | --- | --- | --- | --- | --- | --- | --- | --- |
| ID | x1 | x2 | x3 | x4 | x5 | x6 | x7 | x8 | x9 |
| 1 | 6.7 | 12.7 | 8.1 | 12.4 | 13.6 | 15.9 | 46.8 | 99.3 | Low |
| 2 | 8.3 | 12.7 | 6.6 | 15.4 | 13.6 | 25.8 | 24.2 | 44.6 | High |
| 3 | 6.7 | 12.7 | 8.1 | 15.4 | 6.2 | 25.8 | 46.8 | 44.6 | Low |
| 4 | 8.3 | 12.7 | 8.1 | 15.4 | 13.6 | 15.9 | 24.2 | 99.3 | High |
| 5 | 8.3 | 12.7 | 8.1 | 15.4 | 13.6 | 15.9 | 46.8 | 99.3 | Low |
| 6 | 8.3 | 12.7 | 8.1 | 12.4 | 6.2 | 15.9 | 46.8 | 44.6 | High |
| 7 | 8.3 | 12.7 | 6.6 | 12.4 | 6.2 | 15.9 | 46.8 | 99.3 | High |
| 8 | 6.7 | 12.7 | 6.6 | 15.4 | 6.2 | 25.8 | 46.8 | 99.3 | High |
| 9 | 6.7 | 12.7 | 8.1 | 15.4 | 6.2 | 15.9 | 24.2 | 44.6 | High |
| 10 | 8.3 | 12.7 | 8.1 | 12.4 | 13.6 | 25.8 | 24.2 | 44.6 | Low |
| 11 | 8.3 | 12.7 | 6.6 | 15.4 | 6.2 | 15.9 | 46.8 | 44.6 | Low |
| 12 | 6.7 | 12.7 | 8.1 | 15.4 | 13.6 | 25.8 | 46.8 | 44.6 | High |
| 13 | 8.3 | 12.7 | 8.1 | 12.4 | 6.2 | 25.8 | 24.2 | 99.3 | High |
| 14 | 8.3 | 12.7 | 6.6 | 12.4 | 13.6 | 25.8 | 46.8 | 44.6 | Low |
| 15 | 6.7 | 12.7 | 6.6 | 12.4 | 13.6 | 25.8 | 46.8 | 99.3 | High |
| 16 | 6.7 | 12.7 | 6.6 | 12.4 | 13.6 | 15.9 | 24.2 | 44.6 | High |
| 17 | 6.7 | 12.7 | 6.6 | 15.4 | 13.6 | 15.9 | 24.2 | 99.3 | Low |
| 18 | 6.7 | 12.7 | 8.1 | 12.4 | 6.2 | 25.8 | 24.2 | 99.3 | Low |
| 19 | 8.3 | 12.7 | 6.6 | 15.4 | 6.2 | 25.8 | 24.2 | 99.3 | Low |
| 20 | 6.7 | 12.7 | 6.6 | 12.4 | 6.2 | 15.9 | 24.2 | 44.6 | Low |
| 21 | 6.7 | 15.6 | 8.1 | 12.4 | 6.2 | 25.8 | 46.8 | 44.6 | Low |
| 22 | 8.3 | 15.6 | 6.6 | 15.4 | 6.2 | 15.9 | 24.2 | 99.3 | High |
| 23 | 6.7 | 15.6 | 8.1 | 15.4 | 13.6 | 15.9 | 46.8 | 99.3 | Low |
| 24 | 8.3 | 15.6 | 8.1 | 15.4 | 6.2 | 25.8 | 24.2 | 44.6 | High |
| 25 | 8.3 | 15.6 | 8.1 | 15.4 | 6.2 | 25.8 | 46.8 | 44.6 | Low |
| 26 | 8.3 | 15.6 | 8.1 | 12.4 | 13.6 | 25.8 | 46.8 | 99.3 | High |
| 27 | 8.3 | 15.6 | 6.6 | 12.4 | 13.6 | 25.8 | 46.8 | 44.6 | High |
| 28 | 6.7 | 15.6 | 6.6 | 15.4 | 13.6 | 15.9 | 46.8 | 44.6 | High |
| 29 | 6.7 | 15.6 | 8.1 | 15.4 | 13.6 | 25.8 | 24.2 | 99.3 | High |
| 30 | 8.3 | 15.6 | 8.1 | 12.4 | 6.2 | 15.9 | 24.2 | 99.3 | Low |
| 31 | 8.3 | 15.6 | 6.6 | 15.4 | 13.6 | 25.8 | 46.8 | 99.3 | Low |
| 32 | 6.7 | 15.6 | 8.1 | 15.4 | 6.2 | 15.9 | 46.8 | 99.3 | High |
| 33 | 8.3 | 15.6 | 8.1 | 12.4 | 13.6 | 15.9 | 24.2 | 44.6 | High |
| 34 | 8.3 | 15.6 | 6.6 | 12.4 | 6.2 | 15.9 | 46.8 | 99.3 | Low |
| 35 | 6.7 | 15.6 | 6.6 | 12.4 | 6.2 | 15.9 | 46.8 | 44.6 | High |
| 36 | 6.7 | 15.6 | 6.6 | 12.4 | 6.2 | 25.8 | 24.2 | 99.3 | High |
| 37 | 6.7 | 15.6 | 6.6 | 15.4 | 6.2 | 25.8 | 24.2 | 44.6 | Low |
| 38 | 6.7 | 15.6 | 8.1 | 12.4 | 13.6 | 15.9 | 24.2 | 44.6 | Low |
| 39 | 8.3 | 15.6 | 6.6 | 15.4 | 13.6 | 15.9 | 24.2 | 44.6 | Low |
| 40 | 6.7 | 15.6 | 6.6 | 12.4 | 13.6 | 25.8 | 24.2 | 99.3 | Low |
| 41 | 7.5 | 15.6 | 7.4 | 13.9 | 9.9 | 20.8 | 35.5 | 91.7 | Medium |
| 42 | 7.5 | 12.7 | 7.4 | 13.9 | 9.9 | 20.8 | 35.5 | 91.7 | Medium |
| 43 | 7.5 | 14.2 | 7.4 | 15.4 | 9.9 | 20.8 | 35.5 | 91.7 | Medium |
| 44 | 7.5 | 14.2 | 7.4 | 12.4 | 9.9 | 20.8 | 35.5 | 91.7 | Medium |
| 45 | 7.5 | 14.2 | 8.1 | 13.9 | 9.9 | 20.8 | 35.5 | 91.7 | Medium |
| 46 | 7.5 | 14.2 | 6.6 | 13.9 | 9.9 | 20.8 | 35.5 | 91.7 | Medium |
| 47 | 8.3 | 14.2 | 7.4 | 13.9 | 9.9 | 20.8 | 35.5 | 91.7 | Medium |
| 48 | 6.7 | 14.2 | 7.4 | 13.9 | 9.9 | 20.8 | 35.5 | 91.7 | Medium |
| 49 | 7.5 | 14.2 | 7.4 | 13.9 | 9.9 | 20.8 | 46.8 | 91.7 | Medium |
| 50 | 7.5 | 14.2 | 7.4 | 13.9 | 9.9 | 20.8 | 24.2 | 91.7 | Medium |
| 51 | 7.5 | 14.2 | 7.4 | 13.9 | 9.9 | 20.8 | 35.5 | 91.7 | High |
| 52 | 7.5 | 14.2 | 7.4 | 13.9 | 9.9 | 20.8 | 35.5 | 91.7 | Low |
| 53 | 7.5 | 14.2 | 7.4 | 13.9 | 13.6 | 20.8 | 35.5 | 91.7 | Medium |
| 54 | 7.5 | 14.2 | 7.4 | 13.9 | 6.2 | 20.8 | 35.5 | 91.7 | Medium |
| 55 | 7.5 | 14.2 | 7.4 | 13.9 | 9.9 | 20.8 | 35.5 | 99.3 | Medium |
| 56 | 7.5 | 14.2 | 7.4 | 13.9 | 9.9 | 20.8 | 35.5 | 44.6 | Medium |
| 57 | 7.5 | 14.2 | 7.4 | 13.9 | 9.9 | 25.8 | 35.5 | 91.7 | Medium |
| 58 | 7.5 | 14.2 | 7.4 | 13.9 | 9.9 | 15.9 | 35.5 | 91.7 | Medium |
| 59 | 7.5 | 14.2 | 7.4 | 13.9 | 9.9 | 20.8 | 35.5 | 91.7 | Medium |

mCSA: minimum cross-sectional area
